# Supplementary material for: Comparing the 30-Day Mortality for Hip Fractures in Patients with and without COVID-19: An Updated Meta-Analysis
Source: J Pers Med. 2023 Apr 15;13(4):669. doi: 10.3390/jpm13040669 (PMC10142352; doi:10.3390/jpm13040669)
Supplement: Supplementary file 1 [file jpm-13-00669-s001.zip › jpm-2260674-supplementary.pdf]

Supplementary Table S1. Search strategy (Search on 27 November 2022).

Database	Search term	n
Medline (via OVID)	exp Coronavirus/ OR COVID-19.ti,ab,kw OR coronavirus.ti,ab,kw. OR SARS-CoV.ti,ab,kw. OR SARS-CoV-2.ti,ab,kw. OR 2019-nCoV.ti,ab,kw. OR exp Severe Acute Respiratory Syndrome/<br>AND<br>exp Hip Fractures/ OR exp Femoral Fractures/ OR hip fracture.ti,ab,kw. OR femoral shaft.ti,ab,kw. OR femur shaft.ti,ab,kw. OR periprosthetic.ti,ab,kw. OR femur neck.ti,ab,kw. OR trochanteric.ti,ab,kw. OR intracapsular.ti,ab,kw.	271
Embase	'coronavirus infection'/exp OR 'coronavirus infection' OR 'covid 19' OR 'coronavirus disease 2019' OR 'severe acute respiratory syndrome coronavirus 2'<br>AND<br>'hip fracture' OR 'femur fracture' OR 'femur shaft fracture' OR 'periprosthetic fracture' OR 'femoral neck fracture' OR 'femur trochanteric fracture' OR intracapsular AND fracture	532
Cochrane library	Coronavirus[MeSH] OR Severe Acute Respiratory Syndrome[MeSH] COVID-19 OR severe acute respiratory syndrome<br>AND<br>Hip Fractures[MeSH] OR Femoral Fractures[MeSH] OR hip fracture OR femur shaft fracture OR femur neck fracture OR intracapsular fracture	17
Total		820

Supplementary Table S2. Quality assessments of individual studies.

Study	Selection				Comparability	Outcome / Exposure			Total
	Representativeness of the exposed cohort	Selection of the non-exposed cohort	Ascertainment of exposure	Demonstration that outcome of interest was not present at start of study	Comparability of cohorts on the basis of the design or analysis controlled for confounders	Assessment of outcome	Was follow-up long enough for outcomes to occur	Adequacy of follow-up of cohorts	
Ali (2022)	1	1	1	1	0	1	0	0	5
Arafa (2020)	1	1	1	1	2	1	0	0	7
Barker (2021)	1	1	1	1	0	1	0	0	5
Bayrak (2021)	1	1	1	1	0	1	0	0	5
Beaven (2021)	1	1	1	1	0	1	0	0	5
Biarnes-Sune (2021)	1	1	1	1	0	1	0	0	5
Chan (2022)	1	1	1	1	0	1	0	0	5
Chui (2020)	1	1	1	1	2	1	0	0	7
Clement (2020)	1	1	1	1	0	1	0	0	5
Clough (2020)	1	1	1	1	0	1	0	0	5
Cuthbert (2021)	1	1	1	1	2	1	0	0	7
Dallari (2021)	1	1	1	1	0	1	0	0	5
De (2021)	1	1	1	1	0	1	0	0	5
Egol (2020)	1	1	1	1	2	1	0	0	7
Fadulelmola (2021)	1	1	1	1	0	1	0	0	5
Fell (2021)	1	1	1	1	0	1	0	0	5
Hall (2020)	1	1	1	1	0	1	0	0	5
Hall (2022)	1	1	1	1	2	1	0	0	7
Jimenez-Telleria (2020)	1	1	1	1	2	1	0	0	7
Karayiannis	1	1	1	1	0	1	0	0	5

(2020)									
Kayani (2020)	1	1	1	1	2	1	0	0	7
LeBrun (2020)	1	1	1	1	2	1	0	0	7
Levitt (2022)	1	1	1	1	2	1	0	0	7
Lim (2021)	1	1	1	1	0	1	0	0	5
Macey (2020)	1	1	1	1	0	1	0	0	5
Malik-Tabassum (2020)	1	1	1	1	0	1	0	0	5
Malik-Tabassum (2021)	1	1	1	1	0	1	0	0	5
Mamrelis (2020)	1	1	1	1	0	1	0	0	5
Maniscalco (2020)	1	1	1	1	0	1	0	0	5
Munoz (2020)	1	1	1	1	0	1	0	0	5
Narang (2021)	1	1	1	1	2	1	0	0	7
Oputa (2021)	1	1	1	1	0	1	0	0	5
Rashid (2022)	1	1	1	1	2	1	0	0	7
Segarra (2020)	1	1	1	1	0	1	0	0	5
Sobti (2020)	1	1	1	1	2	1	0	0	7
Thakrar (2020)	1	1	1	1	2	1	0	0	7
Vialonga (2020)	1	1	1	1	2	1	0	0	7
Vives (2020)	1	1	1	1	0	1	0	0	5
Walters (2022)	1	1	1	1	0	1	0	0	5
Wignall (2021)	1	1	1	1	0	1	0	0	5

Wright (2021)	1	1	1	1	2	1	0	0	7
Zamora (2021)	1	1	1	1	0	1	0	0	5

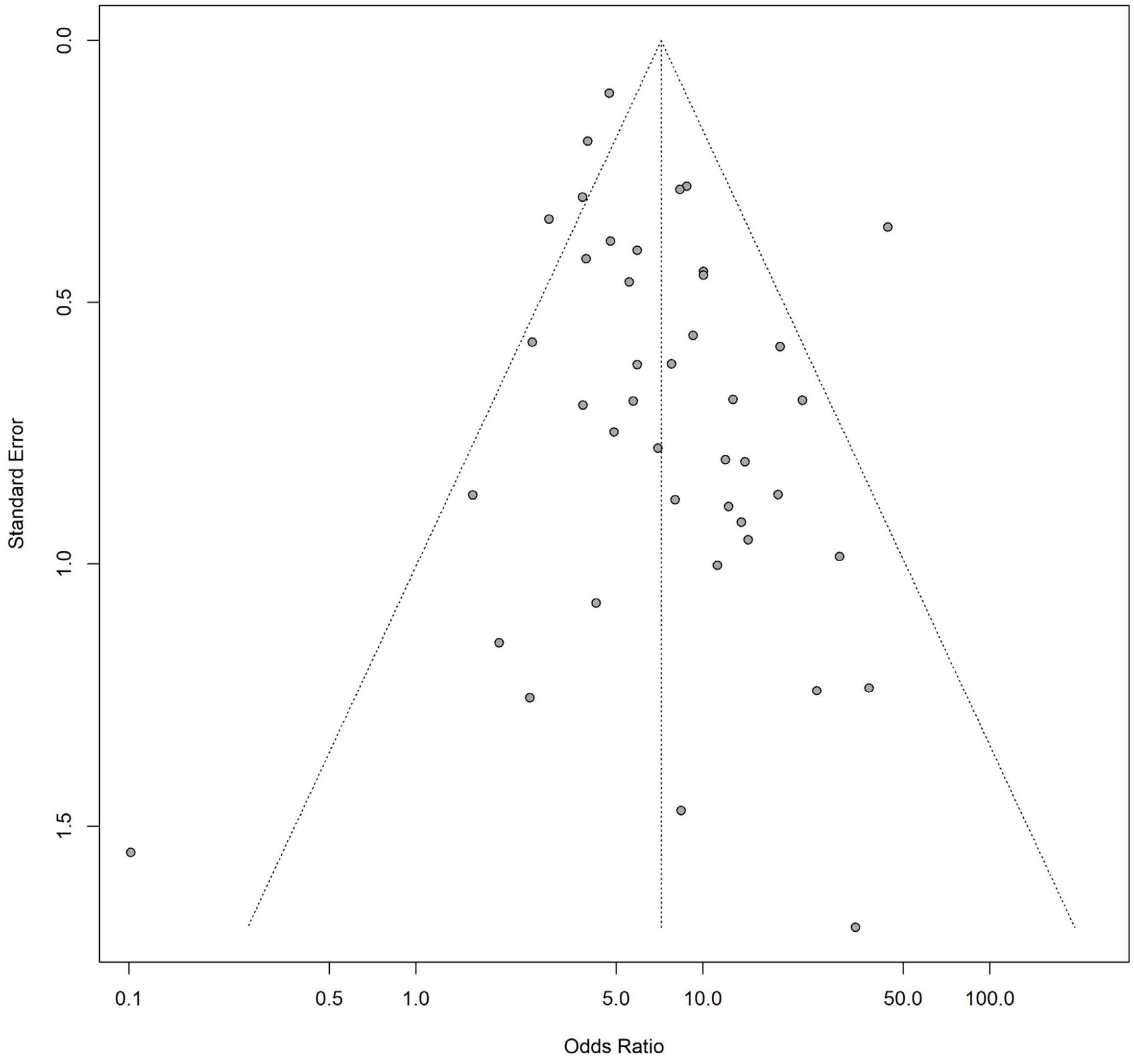

Supplementary Figure S1. Funnel plot for assessment of publication bias.
